# Water temperature modulates multidimensional plastic responses to water flow during the ontogeny of a neotropical fish (*Astyanax lacustris*, characiformes)

**DOI:** 10.3389/fcell.2025.1531162

**Published:** 2025-07-07

**Authors:** Leandro Lofeu, Bianca Bonini-Campos, Tiana Kohlsdorf

**Affiliations:** Department of Biology, FFCLRP, University of São Paulo, São Paulo, Brazil

**Keywords:** BMP4, body shape, body size, ossification, phenotype, plasticity

## Abstract

**Introduction:**

Plastic phenotypes result from multidimensional developmental systems responding to distinct yet simultaneous environmental signals, which may differently affect the magnitude and directions of plastic responses.Concomitant environmental signals during development may result in dominant, synergistic, or even antagonistic phenotypic effects, so that a given condition may amplify or minimize plastic responses to other environmental stimuli. Knowledge on how external information shapes complex plastic phenotypes is essential to predict potential evolutionary trajectories driven by developmental plasticity.

**Methods:**

Here, we manipulate water temperature to evaluate its effects on the well-described phenotypic accommodation of fish growth in the presence of water flow, using the neotropical species *Astyanax lacustris*. We include larval and juvenile ontogenetic stages to examine the interaction between these two environmental signals in plastic responses related to body size and shape, skeleton ossification and gene expression, using *bmp4* as a proxy for ossification pathways.

**Results and discussion:**

Our results demonstrate that water temperature plays a crucial role determining the expression of plastic variation at all dimensions, and effects of water flow were restricted to specific thermal regimes. Combination of high temperature and water flow has a major effect on body shape and unveils unique phenotypic patterns, supporting the prediction that high temperatures can amplify plastic responses to external signals. Specifically, fish raised in the presence of water flow at warmer environments grew faster and ossified earlier, and this condition increased *bmp4* expression levels especially at later developmental stages. Such plastic phenotypes likely involve a functional relationship with swimming performance in running-water environments. Our findings highlight the importance of studying developmental plasticity in complex environments using a multidimensional approach, especially considering increments in water temperatures due to accelerated climate changes that likely impact the fish developmental potential to mitigate environmental changes through plastic responses.

## Introduction

Developmental systems integrate responses to genetic and/or environmental inputs in several dimensions to produce well-suited organisms that are able to survive and reproduce in specific ecological settings (West-Eberhard, 2005; Badyaev, 2009; Duclos et al., 2019). These dimensions interact among each other, and occur both along vertical (e.g., molecules-cells-tissue-organ) and horizontal (i.e., communication among different trajectories and systems within each vertical level) axes (see Duclos et al., 2019). External environmental signals may affect each of these developmental dimensions, shaping the phenotypic outcomes in the ontogenetic landscape along with genes (Duclos et al., 2019). This phenomenon is termed developmental plasticity and designates changes that are frequently permanent in anatomical, physiological and behavioral phenotypes induced by the external environment (West-Eberhard, 2005; Bonini-Campos et al., 2019; Pfennig, 2021; Lofeu et al., 2021; Lofeu et al., 2024). Although developmental plasticity often produces irreversible changes or at least biases subsequent ontogenetic trajectories (West-Eberhard, 2005; Forsman, 2015; Ledón-Rettig and Ragsdale, 2021), literature also recognizes other types of phenotypic plasticity involving responses that may be reversible (Forsman, 2015). Adaptive developmental plasticity allows organisms to handle environmental perturbations by producing functional phenotypes (phenotypic accommodation, see West-Eberhard (2005), which enables population persistence in changing environments (Diamond et al., 2021) and also facilitates adaptive evolution (Hagen, 2008; Hendry, 2016; Uller et al., 2020). Moreover, new complex phenotypes may be revealed by developmental plasticity, which often results in a quick burst of phenotypic diversification (Pfennig and McGee, 2010; Lofeu et al., 2021; Levis and Pfennig, 2021).

Natural environments concomitantly encompass several signals, which may sometimes act in opposite directions in developmental plastic responses (e.g., Kasumovic, 2013; Chevin and Lande, 2015; Groothuis and Taborsky, 2015; Reyes Corral and Aguirre, 2019; Mohanasundaram and Pandey, 2022; Lock et al., 2024). In contrast, some environmental signals seem to amplify the plastic responses to a given stimulus (e.g., Kleiven, et al., 1992; Langerhans et al., 2007; Kasumovic, 2013; Chevin and Lande, 2015; Groothuis and Taborsky, 2015; Lock et al., 2024). Identification of interaction effects among environmental signals presume experimental designs that combine different conditions, although more often studies manipulate one stimulus at a time. For example, in aquatic organisms such as fishes, both temperature and water flow influence development at several dimensions, including gene expression, growth, differentiation and behavior (temperature: Sfakianakis et al., 2004; Witten and Hall, 2015; Riera-Heredia et al., 2018; Shuai et al., 2018; Reyes Corral and Aguirre, 2019; Han et al., 2020; Fey and Greszkiewicz, 2021; Kourkouta et al., 2021; water-flow; Grünbaum et al., 2007; Langerhans, Chapman and DeWitt, 2007; Fischer-Rousseau, Chu and Cloutier, 2010; Fiaz et al., 2012; Witten and Hall, 2015; Kelley et al., 2017; Shuai et al., 2018; Reyes Corral and Aguirre, 2019; Kourkouta et al., 2021). Effects of water flow on fish development are particularly well understood (Langerhans, 2008), although it remains unknown if temperature modulates some of these effects. Water flow often accelerates growth, muscle development (Langerhans, 2008), and chondrogenesis and osteogenesis (Grünbaum et al., 2007; Cloutier et al., 2010; Fiaz et al., 2012), and also induces more streamlined body shapes (Grünbaum et al., 2007; Langerhans, 2008; Reyes Corral and Aguirre, 2019). These plastic responses likely enhance swimming performance in current aquatic environments (Grünbaum et al., 2007; Fiaz et al., 2012), and therefore can be interpreted as phenotypic accommodation (West-Eberhard, 2005). Plastic responses to temperature are also well-known during fish development, and thermal regimes may influence both growth rates and ossification sequences of specific bone sets (see Mabee et al., 2000). Given that changes in water flow might occur in rivers experiencing different thermal regimes (Shuai et al., 2018), temperature effects may overlap and interact with water flow during fish development in three possible outcomes: i) both signals act together, and their interaction induces new responses not observed by only one signal acting individually; ii) one signal has a dominant effect over the other and explains most of the expressed variation; iii) one signal reduces the magnitude of effects of the other. For example, effects of temperature and water flow would be opposite if low temperatures decelerates ossification processes (see Cordova-de la Cruz et al., 2022) while water flow accelerates them (see Fiaz et al., 2012). This complex pattern in the expressed phenotypic variation results from the multidimensional nature of developmental processes along the vertical and horizontal axes described by Duclos et al. (2019). The present study innovates by using an integrative approach in which we evaluate how thermal regimes modulate plastic responses to water flow in several phenotypic traits known to be influenced by both variables. As aforementioned, studies addressing developmental plasticity usually investigate isolated effects of such environmental signals, and here we aim to fill this gap by understanding how effects of one signal (temperature) can influence developmental plastic responses to another signal (water flow).

**FIGURE 1:**
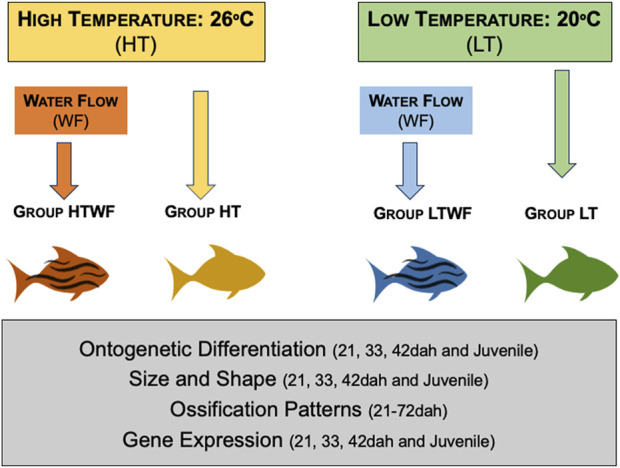
Experimental design. We established two thermal regimes (high temperature = 26°C and low temperature = 20°C) and two conditions of mechanical stimuli (presence and absence of water flow) in each thermal regime. Combination of environmental features resulted in four experimental groups: HT, high temperature and absence of water flow (yellow); HTWF, high temperature and presence of water flow (red), LT = low temperature and absence of water flow (green); LTWF, low temperature and presence of water flow (blue). All groups encompassed quadruplicates (4 aquaria), and we recorded and analyzed 1) ontogenetic differentiation (staging), 2) body size and shape, 3) chondrification and ossification sequences, and 4) *Bmp4* gene expression.

The patterns of plastic responses to specific environmental signals have been described for several fish species (Machado-Schiaffino et al., 2014; Kelley et al., 2017; Härer et al., 2017; O’Dea et al., 2019; Gilbert et al., 2023), but most studies evaluated environmental effects separately, while in nature we expect many signals simultaneously affecting different developmental dimensions. For example, combinations of environmental signals produce different head morphotypes in *M. macrocephalus* fish (Lofeu et al., 2021), and plastic phenotypes encompass developmental changes in several dimensions (Lofeu et al., 2024). In that fish species, some environmental signals seem to have stronger effects in the plastic variation revealed (Lofeu et al., 2021; Lofeu et al., 2024), and we can expect that such effects may also vary along ontogeny. The present study aims to provide a multidimensional perspective about interaction effects of environmental signals on developmental plasticity. Specifically, we investigate the effect of water temperature on plastic responses induced by the presence of water flow, focusing on body size and shape, skeleton ossification, and gene expression during larval and juvenile development of the neotropical fish *Astyanax lacustris* (Characiformes). Several characteristics turn this species an ideal biological system for studies addressing developmental plasticity: these fishes are common for aquarium hobby, as they are small and easy to maintain in captivity; they grow fast and quickly reach sexual maturity, and eggs can be obtained through the year. In addition, previous studies described adaptations to flowing water environments in this species and reported phenotypic plasticity in other species of *Astyanax* (see Costa-Pereira et al., 2016; de França et al., 2024; Reyes Corral and Aguirre, 2019). We manipulated the presence of water flow at two thermal regimes (high temperature = 26°C; low temperature = 20°C) to establish four experimental developmental environments, and quantified plastic responses at different dimensions: 1) growth and differentiation, 2) ossification, 3) gene expression (Figure 1). For this later dimension, we used *bmp4* as a proxy (i.e., an indicator) for ossification pathways, based on the extensive literature describing participation of this gene in processes of bone differentiation (e.g., Ahi, 2016; Li et al., 2021; Wu et al., 2024) and also responses to mechanical stress (e.g., Ikegame et al., 2016; Jang et al., 2016; Dayawansa et al., 2022; Lofeu et al., 2024). Different expression levels of *bmp4* have been identified in plastic responses of fish raised in different developmental environments (Lofeu et al., 2024), which turns this gene an ideal candidate to be a proxy of cellular processes modulating ossification rates in specific environmental conditions. We hypothesize that plastic responses to water flow will be magnified at warmer thermal regimes (see O’Dea et al., 2019). Understanding how water temperature modulates plastic responses to mechanical stimulus is particularly relevant considering accelerated effects of climate changes warming up the aquatic environments (Czernecki and Ptak, 2018).

## Methods

In this study, we aimed to investigate temperature effects in plastic responses triggered by water flow, evaluating multiple interconnected levels: ontogenetic differentiation (staging), growth and size, body shape, ossification rates and gene expression (*bmp4*). As described in introduction, these levels express well-known plastic responses to water flow and also are affected by temperature. Despite being interconnected, we analyze each level as a separated unity, and applied to each dimension the most appropriate methods, considering also specific methodological restrictions for each set of traits.

### Animals and experimental design

We established four experimental developmental conditions that combined presence/absence of water flow (0.10 m/s for 6 h every day) with two thermal regimes (high temperature = 26°C and low temperature = 20°C): 1) no water flow at high temperature (*HT*); 2) presence of water flow at high temperature (*HTWF*); 3) no water flow at low temperature (*LT*); and 4) presence of water flow at low temperature (*LTWF*). The temperatures used correspond to extremes in the thermal range tolerated by this species (Santos et al., 2016; dos Santos et al., 2020), and an interval of six degrees Celsius is sufficient to modify developmental rates of most biological levels investigated here (Sfakianakis et al., 2004; Sfakianakis et al., 2011; Reyes Corral and Aguirre, 2019; Han et al., 2020; Clarkson et al., 2021; Kourkouta et al., 2021). Each experimental condition was assembled in quadruplicate (see detailed description in Supplementary Methods and Supplementary Table S1). We used the species *Astyanax lacustris* as study system. Larvae were acquired from the National Center of Research and Conservation of Continental fishes - CEPTA/ICMBio, Pirassununga, São Paulo, Brazil (Coordinates 21°56′02.8″S 47°22′21.4″W°), a governmental institution established as a genetic bank for conservation of Brazilian fish species. We randomly sampled 1200 larvae that hatched from 600.000 eggs resulting from a mating matrix of 64 females and 128 males (hatching rate of approximately 70%), using a reproductive design that ensured genetic variation similar to wild populations of *A. lacustris*. Twelve days-old larvae (=12 days after hatching, hereafter referred to as 12 dah) were sampled, transported to the lab at University of São Paulo in Ribeirão Preto, São Paulo, Brazil, and acclimatized for 5 days in the same water from the CEPTA at 23°C and constant aeration. We chose to use 12 dah larvae because this stage is the subsequent window after the critical first days of eclosion characterized by high mortality in this species and the individuals were still in the middle of the preflexion stage, which preceeds the developmental window analyzed here. After acclimation time, larvae were distributed in quadruplicates (i.e., four tanks) in each of the four experimental developmental conditions (75 fish per tank, four tanks for each condition, total of 16 tanks). To equally comprise ontogeny in all experimental conditions, we standardized sampling events to occur every 3 days along the entire larval development period up to the juvenile stage. Specifically, at each 3-day interval we randomly collected six specimens from each aquarium (see Supplementary Table S1), in a total of 24 individuals for each experimental condition in each sampling event. We opted to use time in days to standardize sampling events and establish comparison points because this approach avoids bias from metrics like size and ontogenetic stage, which are among the response traits we focus in this study. In addition, strict boundaries among stages are not always clearly demarcated by phenotypic traits in *A. lacustris*, especially within the flexion stage (see Supplementary Methods). By using this approach, we could evaluate which traits differed among experimental groups after fish were maintained for 9 (21dah), 21 (33dah), 30 (42dah) or 60 (72dah) under specific environmental conditions (see further details). Immediately after sampling, we euthanized the animals using an overdose of lidocaine anesthetic solution (1 g/L of water). Two specimens from each aquarium (total of 8 specimens for each experimental group) were stored in RNAlater (Sigma-Aldrich R0901 – Missouri, EUA), and the remaining individuals were fixed in 10% formaldehyde and stored in ethanol 70% for morphological and osteological analyses. The experiments were continued until individuals in all treatments reached the juvenile stage, which happened 60 days after we distributed fish in different environmental conditions. Therefore, we established comparisons based on days after hatching along the experiments, but also compared plastic responses among fish that were at the same stage in the end of the experiments (juveniles). A detailed description is provided in the Supplementary Methods. All procedures were approved by the Ethics Committee for the use of Animals of the Faculty of Philosophy, Sciences and Letters of University of São Paulo in Ribeirão Preto (Protocol #2018.5.310.59.6).

We photographed the specimens in right lateral view by positioning each individual in a petri dish using a brush, using scale and a digital image capture system by a LEICA DMC6200 camera coupled to a LEICA M205 FA stereomicroscope. Pictures were used for ontogenetic staging and also linear and morphometric analyses. Then, we chose three specific sampling time points to better analyze the influence of experimental conditions on ontogenetic differentiation, growth and body shape - 21, 33 and 42 days after hatching, which respectively represented early, middle and late time points of the experiment (∼10 days of difference between each interval) - and defined the juvenile stage as a final point of our analyses (approximately 51dah, depending on the experimental group).

### Ontogenetic staging

To evaluate possible effects of developmental conditions on ontogenetic differentiation (i.e., the rate different ontogenetic stages are established), we staged the specimens sampled from each experimental group following Santos et al. (2016), which describes phenotypic characteristics that separate ontogenetic stages for embryonic, larval and juvenile development in *A. lacustris*. Based on ontogenetic descriptions from Santos et al. (2016), we identified three developmental stages in our sampling: flexion larvae, postflexion larvae, and juvenile. The flexion larvae stage can be subdivided into “early” and “late” according to larvae traits (see Supplementary Methods).

### Body size and shape

We investigated environmentally-induced variation on growth rate and size using linear and geometrics morphometry, and the last was also used to evaluate shape variation (morphospace analysis). For each collection time point (21, 33 and 42dah) we used 16 individuals, except for the juvenile stage, in which we used 64 individuals by group because this was the end of the experiment. Therefore, in addition to the comparisons based on days after hatching along the experiments, we also compared plastic responses among fish that were at the same stage in the end of the experiments (juveniles). Linear measurements of total Body Length (BL) were obtained using ImageJ software version 1.54a (Supplementary Figure S1A). To test for group differences in the average size during ontogeny, we log10 transformed the body length values and performed ANOVA corrected by a Bonferroni post-hoc test.

The same photographed specimens were used for geometric morphometrics analysis. As aforementioned, we based our comparisons on days after hatching, instead of ontogenetic stages. Given that high temperatures accelerate growth (Mabee et al., 2000; see also our results), prior to our shape analyses we performed linear regressions between body height and body length, in order to evaluate if temperature during development affected allometric relationships (see Supplementary Methods). Slopes of allometric relationships did not differ between fish from high and low temperatures raised in the absence of water flow, and slopes were also not different between thermal regimes in fish raised in the presence of water flow (see Supplementary Methods), so we applied morphometric geometrics removing size effects using the pooled-within group approach described in Klingenberg (2016), which is widely applied in studies comparing different species, experimental groups and sex (Sidlauskas et al., 2011; Reyes Corrol and Aguirre, 2019; DeLorenzo et al., 2023; Hetzel and Forsythe, 2023; Pan et al., 2025). We used the software TPSdig version 3.2 (Rohlf, 2005) to position 14 landmarks over homologous points in the larval stages and 17 landmarks in the juvenile stage (anatomic points where landmarks were placed are described in the Supplementary Figures S1B,S1C). We then exported landmark data to the MorphoJ software (Klingenberg, 2011) and performed Procrustes superimposition (“full Procrustes fit”) to remove position, scale (size) and rotation effects, remaining only the shape information that was our trait of interest (see Klingenberg, 2010; Webster and Sheets, 2010). To further remove any remaining allometric effect, we regressed Superimposition-adjusted Procrustes against centroid size, and used the Procrustes residuals for all shape analyses. We implemented these two steps to have confidence that all possible confounding effects of body size were eliminated and only shape information was accessed in the morphospace analysis. A Canonical Variate Analysis was performed to evaluate the morphospace as a function of developmental conditions during ontogenetic windows; using this analysis, we can observe how groups are distributed in the morphospace and which environmental factor explains most of the variation. To test for statistical differences among groups in the morphospace distribution, we performed a one-way ANOVA followed by Bonferroni post-hoc test using the CV scores generated from the Canonical Variate Analysis. We also ran a multivariate regression of Procrustes coordinates (body shape) as a function of centroid size (body size) to evaluate growth rates among experimental groups. These statistical analyses and graphic results were performed in the Prism Software (Swift, 1997). Finally, to test if average shape differs among experimental groups after developing for 61 days in distinct environments and represents new morphotypes, we used the Procrustes coordinates in a Permutational Multivariate Analysis of Variance using Distance Matrices (NPERMANOVA) followed by a Bonferroni *post hoc* test (see Anderson, 2014; Lofeu et al., 2021); this analysis was implemented in the Past Software (Hammer et al., 2001). Similar approaches have been used in previous studies addressing plastic responses in fish to environmental signals manipulated in the lab (Bonini-Campos et al., 2019; Lofeu et al., 2021; Lofeu et al., 2024).

### Ossification patterns of skeletal elements

We evaluated ossification patterns of post-cranium skeletal elements focusing on the time of ossification and the element identity. Specifically, three specimens from each sampling event (three-days intervals along the entire experiment, total of 18 sampling events, see Supplementary Table S1) in each experimental group were stained with Alcian Blue and Alizarin Red, which colors cartilaginous structures in blue and bone structures in red respectively (protocol adapted from Taylor (1985); see Supplementary Methods). Using a digital image capture system in a LEICA DMC6200 camera coupled to a LEICA M205 FA stereomicroscope, we determined the emergence and ossification status of post-cranium skeletal elements as follows: if the element was blue-stained it was classified as cartilaginous, if it was red-stained it was classified as completely ossified. We focused on emergence, chondrification and ossification of the following bone structures: pectoral, caudal, anal, dorsal and pelvic fins, and Weber Apparatus, Vertebral and Supraneural Centers (Supplementary Figure S2).

### Gene expression: *bmp4*

To measure expression levels of *bmp4* (Bone Morphogenetic Protein 4), we preserved fresh samples of post-cranium and caudal fin skeleton in RNAlater (Sigma-Aldrich R0901; Missouri, EUA) and stored at −80°C. We evaluated *bmp4* expression in two ontogenetic windows: 21 and 33dah, respectively representing initial and middle points of the experiments. Larvae at these stages are very small and have low amount of tissue, so we established a pool of two individuals as a unity biological sample. We used three biological samples (each one corresponding to a pool of two individuals) from each experimental group at each ontogenetic stage. Each biological sample was divided into technical triplicates, establishing a total of 9 samples for each group at each ontogenetic stage. The bone structures corresponding to the post-cranium were dissected, mixed, and stored in Trizol (TRI Reagent® RNA Isolation Reagent Sigma Aldrich) for RNA preservation and tissue digestion. We used individuals with similar size and adjusted tissue volume to avoid effects of different volumes between samples. Total RNA was extracted following Trizol RNA-extraction protocol from Invitrogen (T9424; Massachusetts, EUA), and quantified using a Spectrophotometer (NanoVue Plus® Biochrom). The extracted RNA was treated with Invitrogen® Dnase I Kit to remove eventual remaining DNA. Finally, we synthesized cDNA from RNA aliquots (Reverse Transcription) using the Invitrogen® SuperScript® III Kit (Invitrogen 18080051; Massachusetts, EUA).

Expression of *Bmp4* was quantified using real-time PCR (qPCR) and GAPDH as endogenous control. We used primers for *bmp4* and GAPDH from the literature (Casadei et al., 2011; Gross et al., 2016). The qPCR assays were performed using SYBR Green PCR Master Mix (Applied Biosystems 4309155; Massachusetts, EUA) to the final reaction volume of 10 μL and the following cycle conditions: 2 min at 50°C, 10 min at 95°C, followed by 40 cycles of 15 s at 95°C and 1 min at 60°C. All trials were performed in the AriaMx® from Agilent Technologies®.

We analyzed qPCR results using the delta ΔΔCT method, based on Comparative Cycle Thresholding (qCT). Prior to the analyses, we performed an Efficiency (E) test for qPCR experiments, and obtained the following values: *Bmp4 –* E = 0.962 (96.2%), Slope = −3.400; GAPDH–E = 0.981 (98.1%), Slope = −3.282. Data were normalized by log10 transformation. We then calculated the average values of technical triplicates for each biological sample, using 3 biological samples for each experimental group. A one-way ANOVA with Bonferroni post-hoc test was performed to test for differences in quantitative expression among experimental groups. Changes on *bmp4* expression levels as a function of days after hatching was tested using a linear regression (expression levels as response trait and days after hatching as the independent variable). All these analyses were performed in Prism software (Swift, 1997).

## Results

### Temperature modulates the effects of water flow on growth and differentiation

**FIGURE 2:**
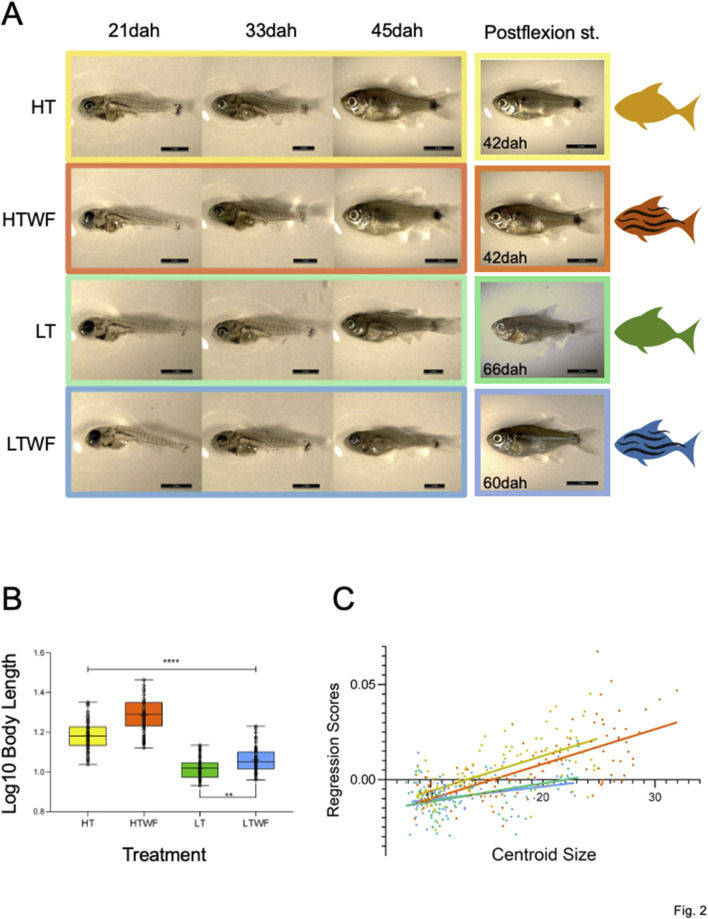
Temperature and water flow affect ontogenetic differentiation and growth. **(A)** Fish phenotypes sampled at 21, 33 and 42dah in each experimental group, and fish phenotypes at the postflexion stage (HT and HTWF fish reached this stage at 42dah, LT reached this stage at 66dah, LTWF reached this state at 60dah). **(B)** Group differences in Body Length (log10 transformed values, *p-values* HT-HTWF/HT-LT/HT-LTWF/HTWF-LT/HTWF-LTWF (****) *< 0.0001*, LT-LTWF (**) = *0.007*). **(C)** Multivariate regression of Procrustes coordinates by Centroid Size (HT: Rsquared = 0.421, F = 77.25, *p-value < 0.0001*. HTWF: Rsquared = 0.488, F = 101.10, *p-value < 0.0001.* LT: Rsquared = 0.164, F = 13.99, *p-value < 0.0004.* LTWF: Rsquared = 0.113, F = 9.436, *p-value < 0.003*)*.* Color codes according to experimental groups: yellow = HT, red = HTWF, green = LT, blue = LTWF.

The presence of water flow accelerated ontogenetic differentiation within each thermal regime (low or high temperatures), but the major factor affecting ontogenetic differentiation was temperature (Figure 2A). At high temperature (HT and HTWF groups) fish already exhibited traits of late flexion larvae 21dah (Supplementary Methods), in addition to better-differentiated eyes, more pigmented body, better developed fins and head when compared to individuals raised at low temperatures, which developed slower regardless of water flow and still resembled early flexion larvae at this sampling event (Figure 2A; see also Supplementary Methods). The same pattern was observed at 33dah: LT and LTWF fish exhibited phenotypic traits of previous flexion substages when compared to HT and HTWF (Figure 2A; see also Supplementary Methods). At 42dah, fish raised at high temperature already exhibited all the features of the postflexion stage: swimming bladder, notochord no longer visible, spread body pigmentation, scales appearing along the body, advanced development of pectoral fins and fin rays, among others (Figure 2A; see also Supplementary Methods). In this condition (i.e., high temperature) it was possible to observe fully-developed juveniles at 51dah, while fish from low temperatures did not reach the juvenile stage until later than 57dah in the LTWF group and later than 60dah in the LT group. Indeed, some individuals raised at low temperatures even did not show phenotypes from late flexion larvae yet at 51dah.

We also observed larger allometric differences between thermal regimes when comparing fish raised in developmental environments with constant water flow, especially because these animals were significantly bigger along larval development when compared to those developed in the absence of water flow (Figure 2B; Supplementary Table S2; Supplementary Figure S3). This observation suggests additive effects on growth between water flow and high temperature, as fish grew faster in warmer environments and water flow also stimulated this process. As a result of this interaction, we identified two contrasts clearly established at 42dah (Figure 2B; Supplementary Table S3; Supplementary Figure S3), which remained during the juvenile stage: 1) fish raised at high temperature (HT and HTWF) significantly larger than those from low temperature (LT and LTWF); 2) fish from water flow conditions (HTWF and LTWF) significantly larger than those raised in the absence of water flow (HT and LT). The multivariate regression of Procrustes coordinates by centroid size confirmed that temperature influenced growth rate, and fish raised at high temperature grew faster than those from low temperature conditions (Figure 2C; Supplementary Table S4).

### Temperature enhances shape responses to water flow and reveals a new morphotype

**FIGURE 3:**
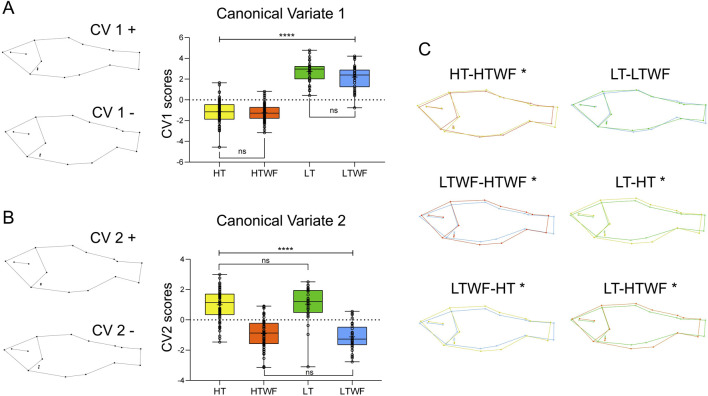
Temperature and water flow influence body shape. **(A)** Canonical Variate 1 (*p-values* HT-LT/HT-LTWF/HTWF-LT/HTWF-LTWF (****) < 0.0001, HT-HTWF/LT-LTWF = 0.999). **(B)** Canonical Variate 2 (*p-values* HT-HTWF/HT-LTWF/HTWF-LT/LTWF-LT (****) < 0.0001, HTWF-LTWF/HT-LT = 0.999). **(C)** Comparisons of average shape among experimental groups at Juvenile stage; (*) indicates different average shape. Color codes according to experimental groups: yellow = HT, red = HTWF, green = LT, blue = LTWF.

The Canonical Variate analysis revealed complex changes in body shape integrating water flow and temperature in two Principal axes (CV 1 and CV 2): in the Canonical Variate 1 (CV1), we detected a major effect of temperature explaining 45% of variation in body shape at 21dah, 71.20% of variation at 33dah, 78.66% at 42dah, and 67.22% at the juvenile stage (Figure 3A; Supplementary Figures S4A,C,E; Supplementary Table S5). In the Canonical Variate 2 (CV2), we detected a major effect of water flow in group positioning (positive and negative values in Figure 3B; Supplementary Figures S4B,D,F; see also Supplementary Table S5). The CV2 explained 36.54% of shape variation at 21dah, 17.39% at 33dah, 12.25% at 42dah, and 22.28% at the juvenile stage. Interestingly, variation explained by water flow decreased in flexion stages of larval development, from 36.54% at 21dah to 12.25% at 42dah, but then increased back to 22.28% at the juvenile stage, which suggests that effects of water flow on body shape increase during the ontogeny of *A. lacustris*.

Temperature effects on body shape were mostly related to body height (i.e., more robust and shorter bodies that are dorsal-ventrally enlarged) in fish raised at high temperatures (HT and HTWF, Figure 3A), while we observed thinner and more elongated bodies that were dorsal-ventrally flattened in fish raised at low temperatures (LT and LTWF, Figure 3A). We also observed that fish from the in HT and HTWF groups were characterized by wider heads that were shortened in the front and had an upturned mouth. At the juvenile stage, fins were positioned closer to the operculum in fish from the LT than in HT groups, suggesting differences in ventral width induced by temperature (Figure 3A). The Canonical Variate 2 was mostly related to water flow and expressed patterns in body shape directly related to swimming function in flow environments (Grünbaum et al., 2007; Langerhans, 2008; Reyes Corral and Aguirre, 2019) that seem exclusive to fish raised in the presence of water flow (HTWF and LTWF conditions). These patterns were already noticeable at 33dah in fish raised from these groups (HTWF and LTWF): a streamlined body, wider dorsal fins, overall more robust and straightforward caudal peduncle, and an upturned rostrum (Supplementary Figures S4D,F). These patterns were even more prominent at the juvenile stage, when fish from the HTWF and LTWF conditions exhibited the typical shape observed in water-current environments: a hydrodynamic streamlined body that is thinner and more fusiform when compared to fish raised in the absence of water flow (HT and LT conditions, see the negative axis at Figure 3B). Moreover, we observed that fish from the HTWF and LTWF groups exhibited a fin shape that is commonly induced as a function of swimming in flow environments, expressing a phenotype characterized by adipose and dorsal fins that are wider (negative axis in Figure 3B). Finally, our results suggest that high temperature promotes shape diversification by enhancing plastic responses to water flow: in high temperatures, fish from the HTWF condition differ from those from HT concerning average shape, while in low temperatures, the presence of water flow is not sufficient to differentiate fish from the LT and LTWF groups regarding average shape (Table 1; Figure 3C). Therefore, interaction between high temperature and presence of water flow induces expression of a new body morphotype that was not observed in environments without water flow (HT group). Comparing shape differences among all groups, it is evident that complex environmental interactions amplified the divergence among fish morphotypes (Table 1; Figure 3C).

**TABLE 1:** NPERMANOVA results testing average shape difference between experimental groups at juvenile stage.

NPERMANOVA				
Permutations	Total sum of squares	Within-group sum of squares	*F*	*p-value*
N = 9,999	0.4022	0.3295	13.89	**0.0001**

Pairwise comparisons: bonferroni-corrected *P*-values				
	HT	HTWF	LT	LTWF
HT	*-*	**0.0006**	**0.0006**	**0.0006**
HTWF	**0.0006**	*-*	**0.0006**	**0.0006**
LT	**0.0006**	**0.0006**	-	0.2532
LTWF	**0.0006**	**0.0006**	0.2532	*-*

HT, high temperature, HTWF, high temperature + water flow, LT, low temperature, LTWF, low temperature + water flow. Significant values (p < 0.05) highlighted in bold.

### Emergence and ossification patterns of post-cranium skeletal elements are affected by temperature and water flow

**FIGURE 4:**
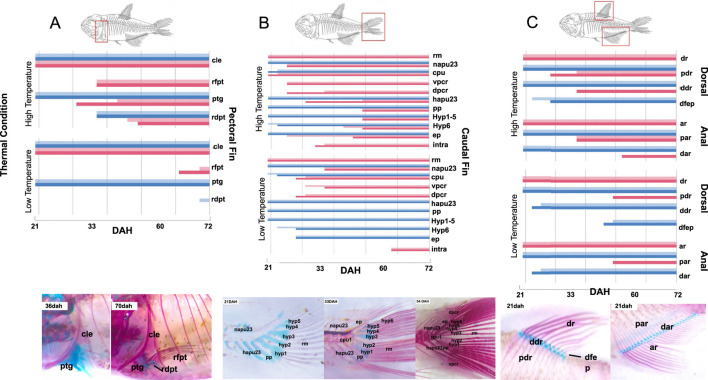
Temperature and water flow affect ossification rate and time. Chondrification and ossification sequence of skeletal elements from **(A)** Pectoral, **(B)** Caudal, **(C)** Dorsal and Anal fins. **(A)** Pectoral fin: pectoral girdle (scapula and coracoid; ptg), cleithrum (cle), pectoral fin rays (rfpt) and pectoral fin radials (rdpt); **(B)** Caudal fin: hypurals (hyp1, hyp2, hyp3, hyp4, hyp5 and hyp6), ural and preural center 1 (cpu1), hemal arches of pu2/pu3 (hapu23), neural arches pu2/pu3 (napu23), Epurais 1 and 2 (ep), Paripural (pp), Main rays (rm), Dorsal procurrent rays (dpcr) and Ventral procurrent rays (vpcr); **(C)** Dorsal fin: Dorsal Rays (dr), Proximal Radials (pdr), Distal Radials (ddr) and Endpiece (dfep); and Anal fin: Anal Rays (ar), Proximal Radials (par) and Distal Radials (dar); pelvic fin: Pelvic Rays (plfr), Radials (plfrd) and Pelvic girdle (plg). The horizontal axes correspond to time, given by days after hatching (dah); the left vertical axes correspond to thermal regime (high or low temperature); the right vertical axes indicate the skeletal element and region. Blue bars correspond to cartilage (bright blue: treatments in the absence of flow [HT and LT], dark blue: treatments with the presence of water flow [HTWF and LTWF]). Red bars correspond to bones (bright red: treatments in the absence of flow [HT and LT], dark red: treatments with the presence of water flow [HTWF and LTWF]).

Overall, chondrification and ossification of most skeletal elements was anticipated in fish raised in higher temperature when compared to those from lower temperature conditions. Interaction of water flow with high temperature (HTWF) resulted in the fastest rates of osteological development, suggesting an additive effect (Figure 4). Low temperatures decelerated ossification rates, but the presence of water flow accelerated ossification of specific elements in this condition, suggesting possible interactions in opposite directions when temperatures are low thermal. Sequence and ossification patterns differed among bone complexes, as further detailed (see also Figure 4; Supplementary Figures S5,S6).

i) Pectoral fin (Figure 4A): the first element to chondrify and ossify in the pectoral fin was the cleithrum (cle), which was completely ossified in all experimental groups at 21dah. The pectoral girdle (ptg), which connects the scapula and coracoid, began to chondrify before 21dah, and was completely ossified by 30dah in fish raised in the HTWF condition, and by 36dah in fish from the HT group. In fish that developed at low temperature (LT and LTWF groups), the pectoral girdle did not ossify before 72dah. The pectoral fin rays (rfpt) appeared by 33dah in fish raised at high temperature, and at 65dah in those developed at low temperature. In low temperature, these structures ossified earlier in the group developed in the presence of water flow (LTWF). The distal pectoral fin radials (rdpt) ossified by 48dah at high temperature, but did not ossify before 72dah at low temperature. Unexpectedly, this structure began to ossify earlier in fish from the group HT than in those from HTWF.
ii) Caudal fin (Figure 4B): The main rays (rm) were the first structure to ossify in the caudal fin. The ural and pre-ural centers (cpu1) ossified first in groups submitted to water flow in both thermal regimes. The same pattern was observed for the dorsal procurrent rays (dpcr), which ossified 10 days earlier in HTWF than in HT group, while the difference in the low temperature groups (LTWF and LT) was approximately 3 days between them. Development of the neural arches of pre-ural centers 2 and 3 (napu23) did not differ between fish raised in the presence or absence of water flow, but these structures ossified near 25dah in fish from high temperature treatments and only by 33dah in those raised at low temperature. We observed a clear interaction between temperature and water flow in the hemal arches of pre-ural centers 2 and 3 (hapu23), which ossified by 25dah in fish from the HTWF group and by 33dah in those from the HT, while in fish from low temperature environments they did not ossify before 72dah. The hypurals (hyp 1-6), epurais (ep) and paripurals (pp) ossified around 50dah in fish raised at high temperature, and did not ossify until 72dah in those from low temperature conditions. Ventral procurrent rays (vpcr) ossified by 25dah and 30dah at high and low temperatures, respectively. Intramuscular (intra) ossified 30 days earlier at high temperature when compared to individuals maintained at low temperature conditions.
iii) Dorsal and anal fins (Figure 4C): dorsal and anal fins showed similar patterns of chondrification and ossification. Dorsal (dr) and anal (ar) rays were the first elements to ossify in both structures, both at high and low temperature conditions. Subsequent ossification was observed in the proximal dorsal and anal radials (pdr and par). We observed a clear influence of water flow in the development of these structures in the low temperature groups, and ossification occurred only in fish from the LTWF group. Distal dorsal and anal rays (ddr and dar) chondrified earlier in fish raised in the presence of water flow (LTWF), but only ossified in the HTWF group. Temperature had an effect also in the dorsal fin endpiece (dfep), which chondrified 20 days earlier in fish from high temperature conditions compared to those from low temperature conditions. In the LTWF group this chondrification was earlier than LT group.
iv) Pelvic fins (Supplementary Figure S5): pelvic fin was the last structure to start ossification. Pelvic fin rays (plfr) ossified by 30dah in fish developed at high temperature conditions, which contrasts with the pattern observed in fish from the LTWF (40dah) and LT (63dah) groups. The pelvic girdle (plg) ossified by 30dah in groups from high temperature conditions. We identified a clear effect of water flow in the low temperature groups: chondrification started by 36dah, and ossification by 65dah in LTWF groups, while in LT groups these started by 45dah and 70dah respectively. Pelvic fin radials (plfrd) ossified by 50dah in high temperature conditions, and did not ossify before 72dah in low temperature conditions. Water flow groups chondrified earlier for both temperatures.
v) Weber Apparatus, Vertebral and Supraneural Centers (Supplementary Figure S6): Most of the structures of the Weber Apparatus and vertebral centers were already ossified by 21dah in fish from high temperature conditions: vertebral centers 1, 2, 3 and 4 (vc14), vertebral centers after post-Weber apparatus (vcpw), and neural (napw) and hemal (hapw) arches. The Supraneural 3 (sn3), post-Weber apparatus supraneurals (snpw) and Parapophyses (parp) were already chondrified by 21dah in fish from the high temperature conditions. Contrary to the pattern observed to the fins, most structures of Weber Complex ossified earlier in fish from HT than those from the HTWF group, while the pattern was inverse in LT and LTWF groups. Neural arcs of the Weber apparatus (na34) ossified by 25dah in high temperature and 36dah in low temperature. Supraneural 3 (sn3) ossified by 33dah in fish from the HT condition, 50dah at HTWF, 63dah at LTWF, and by 72dah at LT condition. Paripurals (parp) ossified 30 days earlier in groups from high temperature conditions than those from low temperature. Post Weber appliance (snpw) supraneurals ossified by 50dah at high temperature and by 72dah at low temperature.

### Water flow affects *Bmp4* expression levels during skeletal ontogeny

**FIGURE 5:**
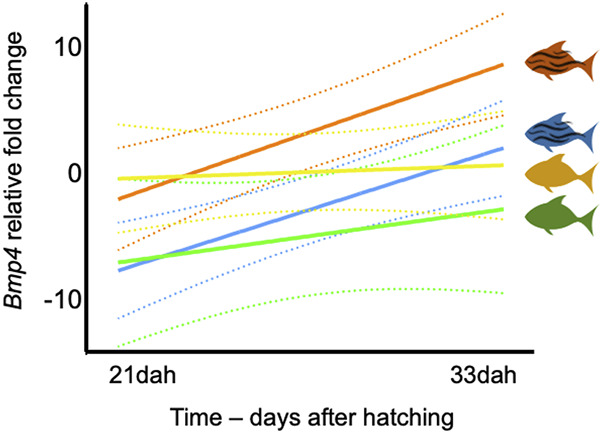
Expression of *bmp4* is mostly affected by temperature. Regression of *bmp4* expression levels in two sampling events: 21dah and 33dah. HT: Rsquared = 0.056, F = 0.2382, *p-value* = 0.651. HTWF: Rsquared = 0.870, F = 26.94, *p-value* = 0.006. LT: Rsquared = 0.279, F = 1.554, *p-value* = 0.280. LTWF: Rsquared = 0.863, F = 25.29, *p-value* = 0.007. Color codes according to experimental groups: yellow = HT, red = HTWF, green = LT, blue = LTWF. Relative fold changes in the vertical axis indicate how much the *bmp4* expression level differs from the calibrator group (HT) after all samples were normalized by the housekeeping gene GAPDH; dashed lines = confidence intervals (95%).

At 21dah, we identified temperature as a major factor affecting *bmp4* expression in analyzed samples, with higher expression values measured in groups developed at higher temperature (HT and HTWF) when compared to those from lower temperature conditions (LT and LTWF). However, significant differences were only identified between HT and LT and LTWF groups (Supplementary Figure S7; Supplementary Table S6). At 33dah, we also identified effects of water flow in *bmp4* expression levels, especially in the HTWF group, which exhibited higher expression values than all other groups (Supplementary Figure S7; Supplementary Table S6). Expression levels of *bmp4* increased with time (days after hatching) both in fish from HTWF and LTWF conditions, but not in the HT and LT groups, suggesting effects of water flow on *bmp4* expression during larval ontogeny (Figure 5; for specific data for each condition, see also Supplementary Figure S8; Supplementary Table S7).

## Discussion

Developmental plastic responses often produce complex highly-integrated phenotypes (Westneat et al., 2019) and encompass multidimensional processes in which different structures at several biological levels interact to determine the ontogenetic landscapes (Duclos et al., 2019). In each dimension, a myriad of environmental signals may concomitantly determine and modulate trajectories in landscapes (Duclos et al., 2019). Moreover, distinct environmental signals may differ in the strength of their effects on development, and may interact in an additive, synergistical or even antagonist way. Understanding how distinct yet simultaneous environmental signals shape plastic responses is essential to predict how developmental plasticity may affect trajectories of phenotypic evolution in specific lineages. This is especially relevant in the current scenario of global warming and accelerated climate changes. In the present study, we manipulated water temperature to evaluate multidimensional plastic responses to water flow during larval and juvenile ontogeny of the fish *Astyanax lacustris*. Our results provide evidence of interaction effects between temperature and water flow in growth, ossification and gene expression in this tropical fish.

### Thermal regimes impact several levels of ontogenetic development, and effects of water flow are modulated by temperature

When studying developmental plasticity in complex environments, a very relevant question is whether a given variable has a major effect over the others (Westneat et al., 2019), and if there is a driver determining the magnitude and direction of plastic responses to other variables (see Lofeu et al., 2021). In the system used in the present study, manipulation of two environmental variables simultaneously suggests that effects of temperature on fish development seem more prominent than those of water flow in most of the processes evaluated. Specifically, we identified two remarkably distinct phenotypic groups as a function of thermal regimes, especially regarding body size and external differentiation during ontogenety (early flexion, flexion, postflexion larvae, and juvenile stages). Development at higher temperature resulted in larger, well-developed and differentiated fish, which contrasts with the smaller and less-differentiated fish observed in colder thermal regimes. Despite reports of accelerated bone development in fish raised in the presence of water flow (see Cloutier et al., 2010; Fiaz et al., 2012), we observed that temperature is actually a major effect that overlaps mechanical stimulus and defines ossification time of skeletal elements in *Astyanax lacustris*, decelerating ossification at colder environments. Given that mechanical stress by differential exercise seems to induce robust ossification in fish (Fiaz et al., 2012; Lofeu et al., 2024), we interpret that cold environments may decelerate bone ossification regardless the presence of water flow. Nonetheless, phenotypic effects of water flow were still noticeable in the groups developed in the presence of water flow, as this factor accelerated chondrification and ossification of specific elements related to swimming. At warmer thermal regimes, water flow has an additive effect to high temperature, maximizing growth and ossification (see Grünbaum et al., 2007; Cloutier et al., 2010; Fiaz et al., 2012).

### Temperature and water flow elicit expression of shape plasticity - higher temperature reveals a “water-flow plastic morphotype”

We identified that thermal regimes explain most of the variation in body shape during all ontogenetic stages we studied in *A. lacustris*, and water flow seems to interact together with temperature producing unique morphotypes. Rather than only acting additively by enhancing a general phenotypic pattern, such as body size or bone ossification, combination of water flow with high temperature reveals phenotypes that are not induced by one isolated environmental variable. These ‘water-flow plastic morphotypes’ are illustrated by the shape tendencies in the CV2 at 33 days after hatching, which become even more evident in late larval and juvenile stages; these resemble known shape changes related to water flow-associated fish phenotypes (see Grünbaum et al., 2007; Langerhans, 2008; Reyes Corral and Aguirre, 2019). In *A. lacustris*, it is possible to identify four morphotypes, two associated with thermal regimes, and two arising from the interaction between thermal regime and water flow. The warm thermal regime used here apparently enhanced specific plastic responses to water flow in the HTWF group that has not been reported in natural populations, suggesting the potential for variation in *A. lacustris* revealed by specific combinations of environmental factors and may be a source of adaptive plasticity (see Schlichting, 2008; Bonini-Campos et al., 2019; Lofeu et al., 2021). The aquaculture population from which we obtained the hatchlings is maintained in a controlled environment with stable temperature near 23°C and has never been exposed to water flow, and even in nature *A. lacustris* is usually associated to lentic water environments (da Silva et al., 2021). Therefore, the shape morphotypes revealed by our experimental environments in the lab may be interpreted as “novel phenotypes” when compared to the aquaculture system or to natural populations of this species. Although our study did not address eventual differences in oxygen concentration derived from temperature manipulation, we recognize lower concentrations of dissolved O2 in the water (see Rutjes et al., 2009) might partially explain wider heads in *Astyanax* fish from the high-temperature groups (HT and HTWF). In other fish species, similar phenotypic patterns have been induced by hypoxia derived from high temperatures, and the larger heads observed in fish raised in such conditions may harbor larger gills (Collyder, 2003; Rutjes et al., 2009; Lema et al., 2019). These observations highlight the benefits of gathering as much information as possible about the development environment in future studies investigating plasticity as an inducer of new phenotypes in ecologically relevant contexts. Our results illustrate how diverse can be the ontogenetic responses to complex ecological contexts, and are also coherent with metanalyses suggesting lower phenotypic means for morphological traits in fishes from colder water environments (O’Dea et al., 2019). Therefore, warmer environments may enhance phenotypic variation (see O’Dea et al., 2019) and also enable expression of new plastic phenotypes, eventually by magnifying phenotypic responses to other concomitant environmental signals (Westneat et al., 2019; Potts et al., 2021; Lock et al., 2024). The plastic potential discussed here may be a source of adaptive responses to global warming (O’Dea et al., 2019) and should be further investigated in natural populations. Temperature could be an environmental modulator of plastic responses to other environmental factors by facilitating, biasing or even restricting the expression of new plastic phenotypes depending on which factors are interacting with temperature.

Why temperature has a dominant effect on all ontogenetic processes investigated here? And why water flow alone is not able to induce distinct morphotypes at lower temperatures? The answer it is not so clear, but it probably resides on the role of temperature in the cell metabolism and growth (Guderley, 2004; Viadero, 2005; Lema et al., 2019; Fey and Greszkiewicz, 2021), producing overall effects in the organism. Temperature affects efficiency of food intake and conversion (Kinne, 1960) and expression levels of Growth Hormone (GH, see Deane and Woo, 2009). Even knowing that water flow may influence growth dynamics in several tissues, especially cartilage and bones (Fiaz et al., 2012), and also affect body shape (Rutjes et al., 2009; Fischer-Rousseau et al., 2010), responses to flow stimulus may be directly modulated by temperature at metabolic and cellular levels. In fact, temperature affects cartilage and bone development (Blaxter, 1992; Mabee et al., 2000; Hall, 2005; Cubbage and Mabee, 1996; Georgakopoulou et al., 2010; Sfakianakis et al., 2011), and shape emerges as the summatory of different levels (Reyes Corral and Aguirre, 2019), including osteological elements.

### Ontogenetic changes induced by water flow suggest developmental responses connecting form to function

We identified a major effect of temperature on all traits investigated here, but water flow also seems to define the appearance of some phenotypic patterns in *A. lacustris* that are known for their functional e adaptive roles related to swimming performance (Fischer-Rousseau et al., 2010; Shuai et al., 2018). Phenotypic patterns observed in fish raised in the presence of water flow involve wider dorsal, adipose and pectoral fins, and longer and streamlined thinner fusiform bodies (Langerhans, 2008; Reyes Corral and Aguirre, 2019), which enhance fish swimming manouvrality in the water, consequently incluencing foraging, scape, and predation activities by fish. The presence of water flow accelerates chondrification and ossification in several fish species (see Cloutier et al., 2010; Fiaz et al., 2012), and we observed a similar pattern in *A. lacustris* (see results for HTWF and LTWF groups). The functional bridge between accelerated ossification and water flow if furthermore sustained by increased expression levels of *bmp4* in fish raised in the presence of water flow, especially at 33 days after hatching (see also Parsons and Albertson, 2009). Together with other members of bmp gene family, *bmp4* is involved in several processes related to bone formation, proliferation and remodeling (Ahi, 2016; Li et al., 2021; Wu et al., 2024). Changes along ontogenetic time in *bmp4* expression levels suggest a complex interaction of temperature and water flow modulating *bmp4* expression, especially considering that each factor isolated can affect expression levels of this gene (Han et al., 2020; Clarkson et al., 2021; Li et al., 2021). More interesting, we identified a clear increase of *bmp4* expression levels from 21 to 33 days after hatching in fish from the water flow conditions (HTWF and LTWF). Although here we do not address a direct relationship between genotype and phenotype, empirical studies demonstrated that higher expression levels of *bmp4* enhance chondrogenesis and osteogenesis, also contributing for biomechanical performance of skeletal elements during fish development (Terai et al., 2002; Albertson et al., 2005; Parsons and Albertson, 2009; Li et al., 2021). Mechanical stress and exercise can affect expression levels of *bmp4* (Young and Badyaev, 2007; Ikegame et al., 2016; Jang et al., 2016; Dayawansa et al., 2022; Lofeu et al., 2024), and we interpret changes in *bmp4* expression associated with the presence of water flow in our experiments and the phenotypic patterns induced in *A. lacustris* as indicators of functional plastic responses also at the cellular level. To conclude, our results reiterate the benefits of performing experiments using several relevant environmental signals concomitantly, in order to address the potential of developmental plasticity to induce new phenotypes, an assumption of the ‘Plasticity-led evolution Hypothesis’ (Levis and Pfennig, 2021). Some phenotypes may only be expressed under specific combinations of environmental signals (see also Lofeu et al., 2021; 2024), and plastic responses may constitute a key factor determining extinction or adaptation processes in changing environments.

## Data Availability

The datasets presented in this study can be found in online repositories. The names of the repository/repositories and accession number(s) can be found in the article/Supplementary Material.
