# Supplementary material for: Water temperature modulates multidimensional plastic responses to water flow during the ontogeny of a neotropical fish (Astyanax lacustris, characiformes)
Source: Front Cell Dev Biol. 2025 Jul 7;13:1531162. doi: 10.3389/fcell.2025.1531162 (PMC12277331; doi:10.3389/fcell.2025.1531162)
Supplement: Supplementary file 1 [file DataSheet2.pdf]

SUPPLEMENTARY METHODS

Maintenance during experimental conditions

After the acclimatization period, the individuals were distributed in aquariums measuring 40x16x30 cm, with a water volume of 6L. The fish were divided into four aquariums for each development condition (quadriplicates). The 16 aquariums (development conditions in quadruplicate) were coupled to a system with constant aeration and mechanical (acrylic blanket), chemical (activated carbon) and biological (ceramic for fixing bacterial colonies) filter with a flow rate of 1000 L/h. The temperature of each aquarium was confirmed weekly throughout the experiment. Temperature control was performed using Atman AT-200 thermostats set at 28°C, fixed in water filtering tanks for high temperature development conditions: keeping the water temperature by 26ºC ± 1ºC. For low temperature development conditions room was maintained at 18°C, keeping the water by 20ºC ± 1ºC. The internal Water Flow of the aquariums for WF groups, was maintained with pumps of 200 L/h connected to a system regulated by a timer, turned off during the night and alternated during the day, in which they alternated between remaining on for one hour and then off for a subsequent hour, totaling 6 hours of flow daily. Average velocity of 0.10 m/s. The water used to start the system was taken from the eutrophic lake Monte Alegre (Silvia, 1999) located on the USP campus in Ribeirão Preto (21°11'S 47°43'W), collected with the aid of 6-liter gallons and transported to the animal facility with authorization from the Campus City Hall. Daily, the system water was topped up using tap water, after a decanting process for at least two days and conditioned in the appropriate proportion of Prime Seachem®, to neutralize ammonia, assist in the removal of nitrate, nitrite, chlorine, chloramine and heavy metals. system make-up water. The fish were fed four times a day, at the beginning and at the end of the morning and afternoon, with commercial Lambari mash food containing 44% crude protein.

Linear Regressions testing for differences in allometric growth

Given that temperature affected growth rates (see results), we decided to test if allometric relationships differed among thermal regimes, especially in traits we identified plastic responses in the shape analyses using morphometric geometrics based on days after hatching, instead of stage. To do that,

we performed linear regressions of body height (the most prominent difference in body shape among conditions) by body length, testing for differences in slope between thermal regimes separately for fish raised in the presence of water flow and for those raised in the absence of water flow. As illustrated by the following graphs, the slope of allometric curves was similar among fish raised at different temperatures in the absence of water flow (Figure 1: F = 0.7079, P=0.4006), a similar result to that observed among fish raised in the presence of water flow (Figure 2: F = 3.822, P=0.0513.).

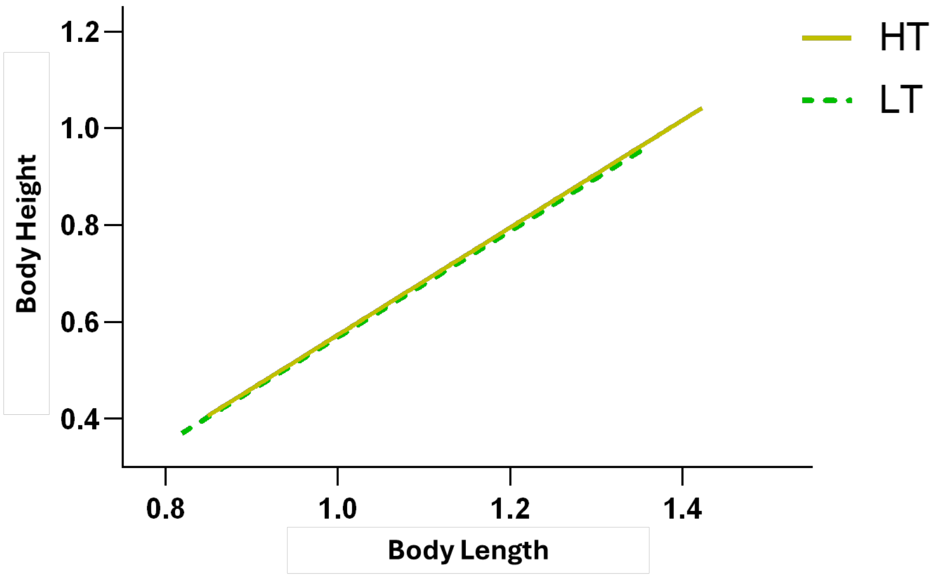

Figure 1: regression of Body Height by Body Length for all animals sampled along the development in the HT and LT groups.

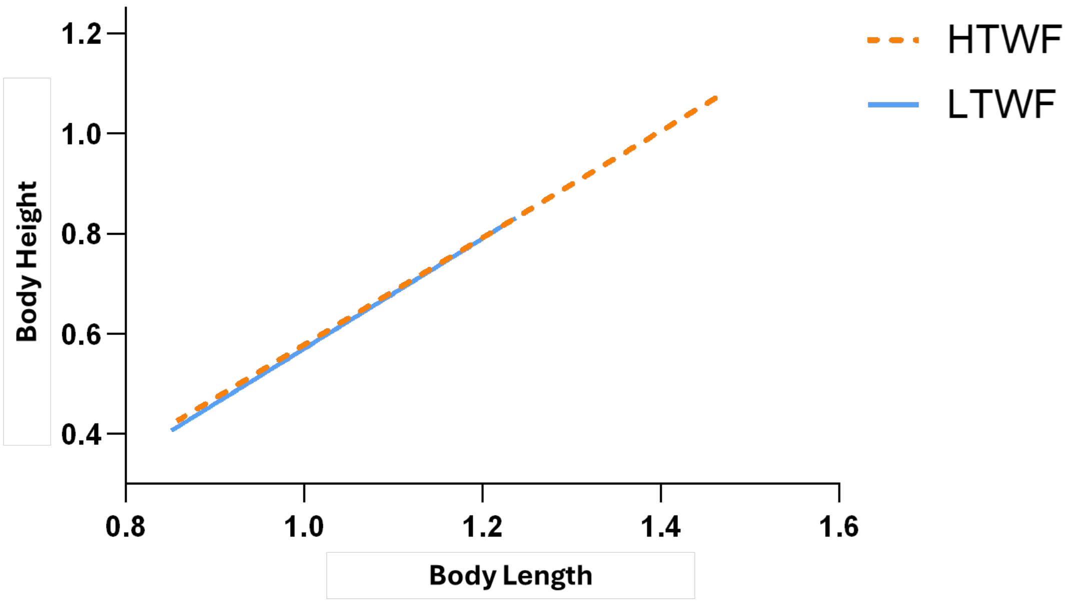

Figure 2: regression of Body Height by Body Length for all animals sampled along the development in the HTWF and LTWF groups.

Staging of *Astyanax lacustris*

A detailed description of phenotypic traits used to identify ontogenetic stages in *Astyanax lacustris* is presented in figure 3; staging was based on based on Santos et al. (2019). As it can be noticed from the picture, strict boundaries among stages are not always clearly demarcated by these traits, especially within the flexion stage, which also justifies the comparisons based on days after hatching instead of those exclusively based on staging.

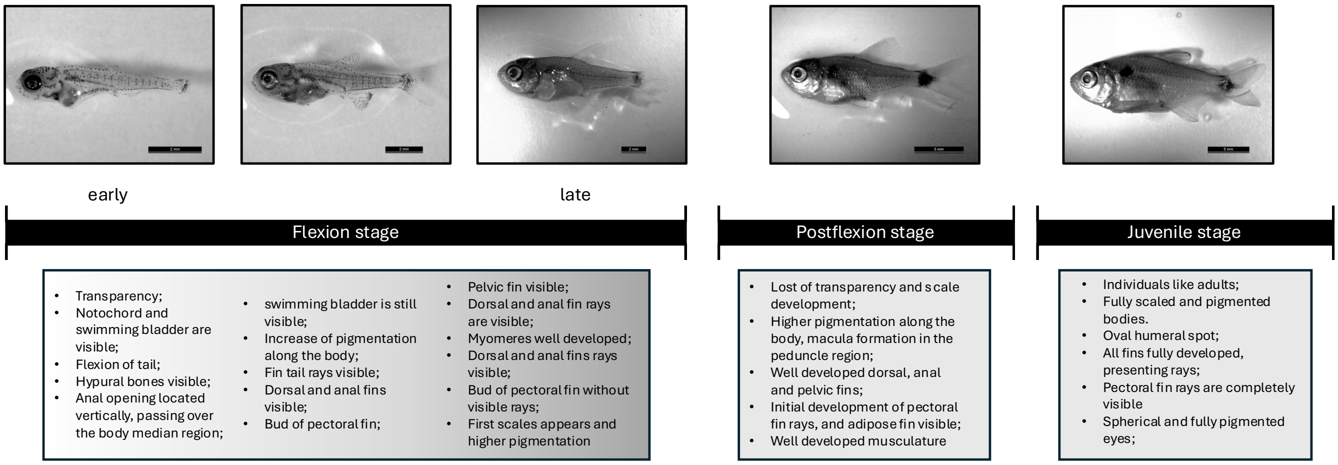

Figure 2: Staging of *Astyanax lacustris* at Postflexion and Juvenile stages; flexion stage was subsequently divided into early and late. The principal hallmarks of each stage are shown in the box below each stage bar. For flexion stage, different tons of grey indicate early (clear) to late (darker grey). Above stage bars, photos of specimens from HT treatment, showing the principal features of each stage.

Alizarin Red and Alcian Blue Protocols (modified from Taylor & Van Dyke, 1985 and Westerfield, 2007).

Fixation

10% formaldehyde buffered **24 hours**
(10 ml formaldehyde; 90 ml distilled water; 0.65 g NaHPO4; 0.4 g NaH2PO4)
Ethanol 50/50 **24 hours**
(50 ml absolute ethanol; 50 ml distilled water)
Ethanol 70/30 **24 hours**
(70 ml absolute ethanol; 30 ml distilled water)
Absolute Ethanol **24 hours**
(100 ml absolute ethanol)

Preparation

24 hours xylene (Only for specimens larger than 10 mm)

Cartilage staining

Alcian Blue **24 hours**
(30 ml acetic acid CH3COOH; 70 ml absolute alcohol; 0.02 g Alcian Blue)
Borax 25/75 **2 hours**
(25 ml saturated solution of borax and distilled water; 75 ml absolute alcohol)
Borax 50/50 **2 hours**
(50 ml saturated solution of borax and distilled water; 50 ml absolute alcohol)
Borax 75/25 **2 hours**
(75 ml saturated solution of borax and distilled water; 25 ml absolute alcohol)
Borax 100 **2 hours**
(100 ml saturated solution of borax and distilled water)

Pigmentation Removal

Hydrogen Peroxide – 24-Hour UV Light Exposure
(10 ml hydrogen peroxide H2O2; 90 ml distilled water; 0.45 g KOH)

Tissue Removal

Trypsin 1 to 20 hours
(35 ml saturated solution of Borax and distilled water; 65 g distilled water; 1 g Trypsin)
*During this step, keep at 35°C. Observe every 15 minutes until specimens are 60% translucent.

Bone Coloring

Alizarin **24 hours**
(100 ml distilled water; 0.045 g potassium hydroxide KOH; 0.015 g Alizarin)
Wash in distilled water and remove eyes and scales

Dehydration

Potassium Hydroxide **24 hours**
(90 ml distilled water; 1.8 g potassium hydroxide KOH)
Glycerin 40/60 **12 hours**
(40 ml of glycerin; 60 ml of distilled water; 0.3 g KOH)
Glycerin 70/30 **12 hours**
(70 ml of glycerin; 30 ml of distilled water; 0.15 g KOH)

Preservation

Glycerin
(100 ml of glycerin; 0.01 g Thymol)
