# Supplementary material for: Water temperature modulates multidimensional plastic responses to water flow during the ontogeny of a neotropical fish (Astyanax lacustris, characiformes)
Source: Front Cell Dev Biol. 2025 Jul 7;13:1531162. doi: 10.3389/fcell.2025.1531162 (PMC12277331; doi:10.3389/fcell.2025.1531162)
Supplement: Supplementary file 2 [file DataSheet3.pdf]

Supplementary Figures

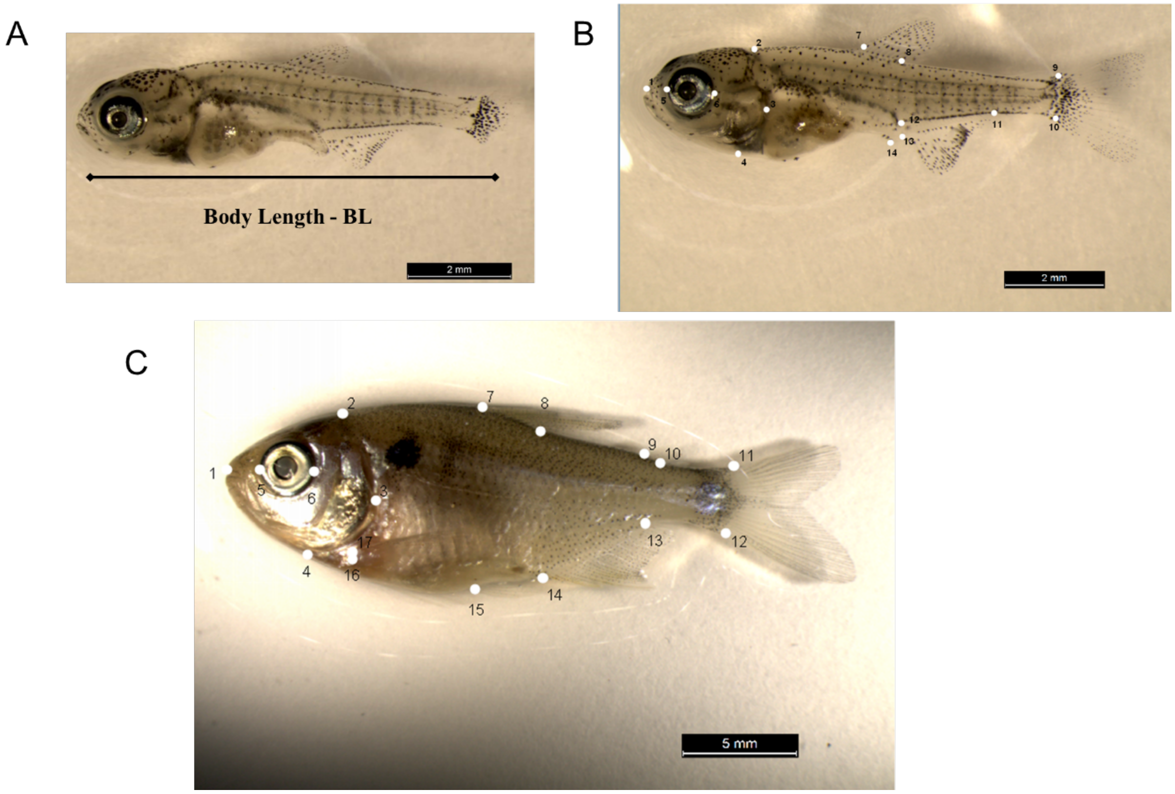

Supplementary Figure 1: Body Length and Landmarks used in morphometric analysis. A) Body Length (BL), taken from mouth to end of caudal peduncle, using ImageJ Software version 1.54a. B) 14 landmarks in larval stage: 1- mouth, 2- end of head, 3-posterior edged of opercule, 4- ventral opercular isthmus, 5- front of eye, 6- end of eye,7- front of dorsal fin, 8- end of dorsal fin, 9- superior end of caudal peduncle, 10- inferior end of caudal peduncle, 11- end of anal fin, 12- front of anal fin, 13- posterior anal opening, 14- anterior anal opening. C) 17 landmarks in larval stage: 1- mouth, 2- end of head, 3-posterior edged of opercule, 4- ventral opercular isthmus, 5- front of eye, 6- end of eye, 7- front of dorsal fin, 8- end of dorsal fin, 9- front of adipose fin, 10- end of adipose fin, 11- superior end of caudal peduncle, 12- inferior end of caudal peduncle, 13- end of anal fin, 14- front of anal fin, 15- insertion of pelvic fin, 16- inferior insertion of pectoral fin, 17- superior insertion of pectoral fin.

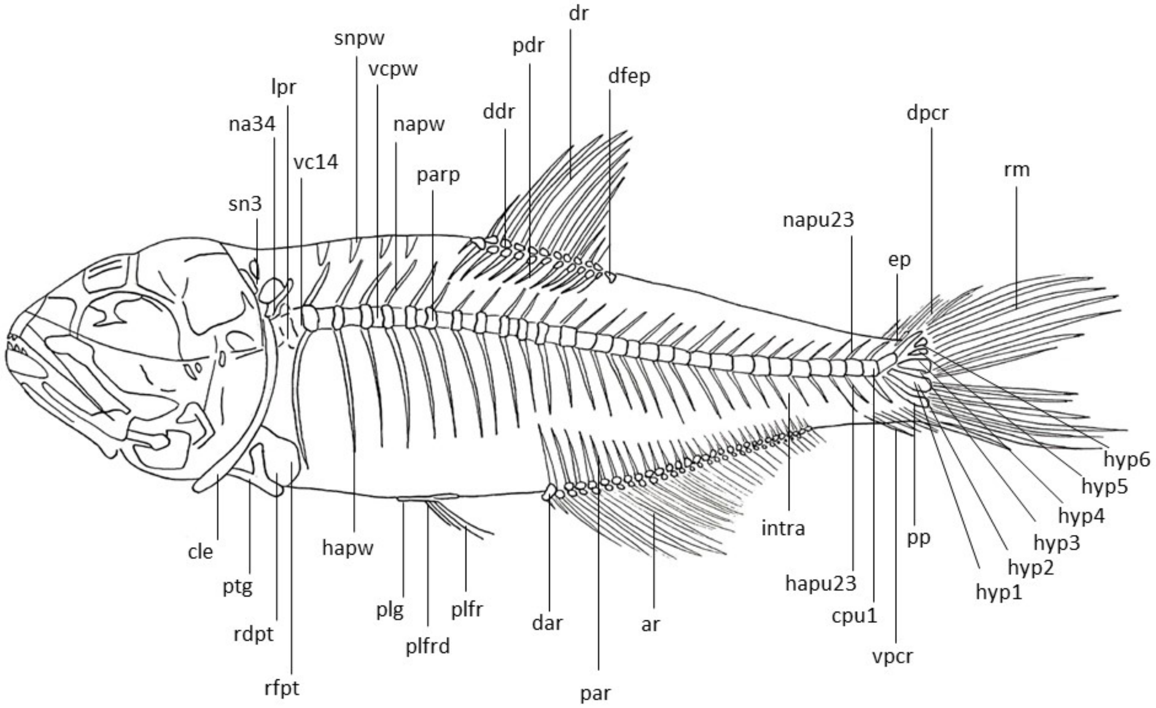

Supplementary Figure 2: Skeletal elements used to compare chondrification and ossification sequence among experimental groups. **Weber Apparatus:** composed of lateral processes (lpr), neural arches of vertebrae 3 and 4 (na34), vertebral centers 1, 2, 3 and 4 (vc14), and supraneural 3 (sn3); **post-Weber (pw) Apparatus elements:** vertebral Centers (cvpw), Hemal Arches (hapw), Neural Arches (napw), Parapophyses (parp), Supraneural (snpw) and Intramuscular (intra); **pectoral fin:** pectoral girdle (scapula and coracoid; ptg), cleithrum (cle), pectoral fin rays (rfpt) and pectoral fin radials (rdpt); **caudal fin:** hypurals (hyp1, hyp2, hyp3, hyp4, hyp5 and hyp6), ural and preural center 1 (cpu1), hemal arches of pu2/pu3 (hapu23), neural arches pu2/pu3 (napu23), Epurais 1 and 2 (ep), Paripural (pp), Main rays (rm), Dorsal procurrent rays (dpcr) and Ventral procurrent rays (vpcr); **dorsal fin:** Dorsal Rays (dr), Proximal Radials (pdr), Distal Radials (ddr) and Endpiece (dfep); **anal fin:** Anal Rays (ar), Proximal Radials (par) and Distal Radials (dar); **pelvic fin:** Pelvic Rays (plfr), Radials (plfrd) and Pelvic girdle (plg).

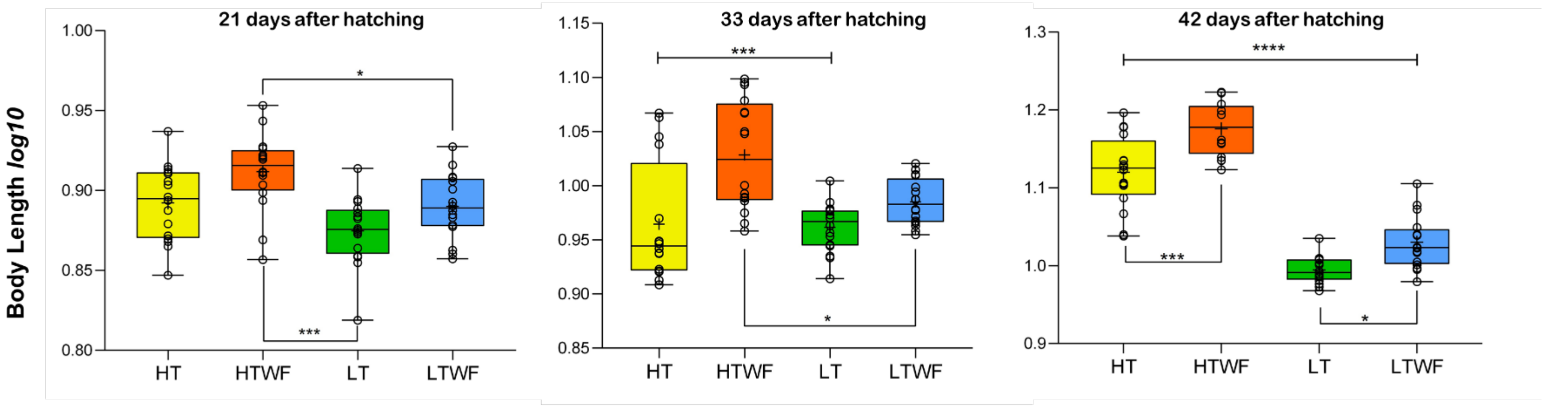

Supplementary Figure 3: Body Length, log10 transformed values, between experimental groups at 21, 33, and 42 days after hatching. 21 days after hatching: *p-values* HTWF-LTWF (*): *0.039*, LT-HTWF (***): *0.0001*. 33 days after hatching: *p-values* HT-LT/HT-LTWF/HTWF-LT/HTWF-LTWF (*): *0.022*, (***) HT-HTWF/HT-LT: *0.0001*. 42 days after hatching: E) *p-values* HT-LTWF/HT-LT/HTWF-LT/HTWF-LTWF (****): < *0.0001*, HT-HTWF (***): *0.0002*, LT-LTWF (*): *0.031*. Mustard: HT (Hight Temperature), Orange: HTWF (Hight Temperature Water Flow), Green: LT (Low Temperature), Blue: LTWF (Low Temperature Water Flow)

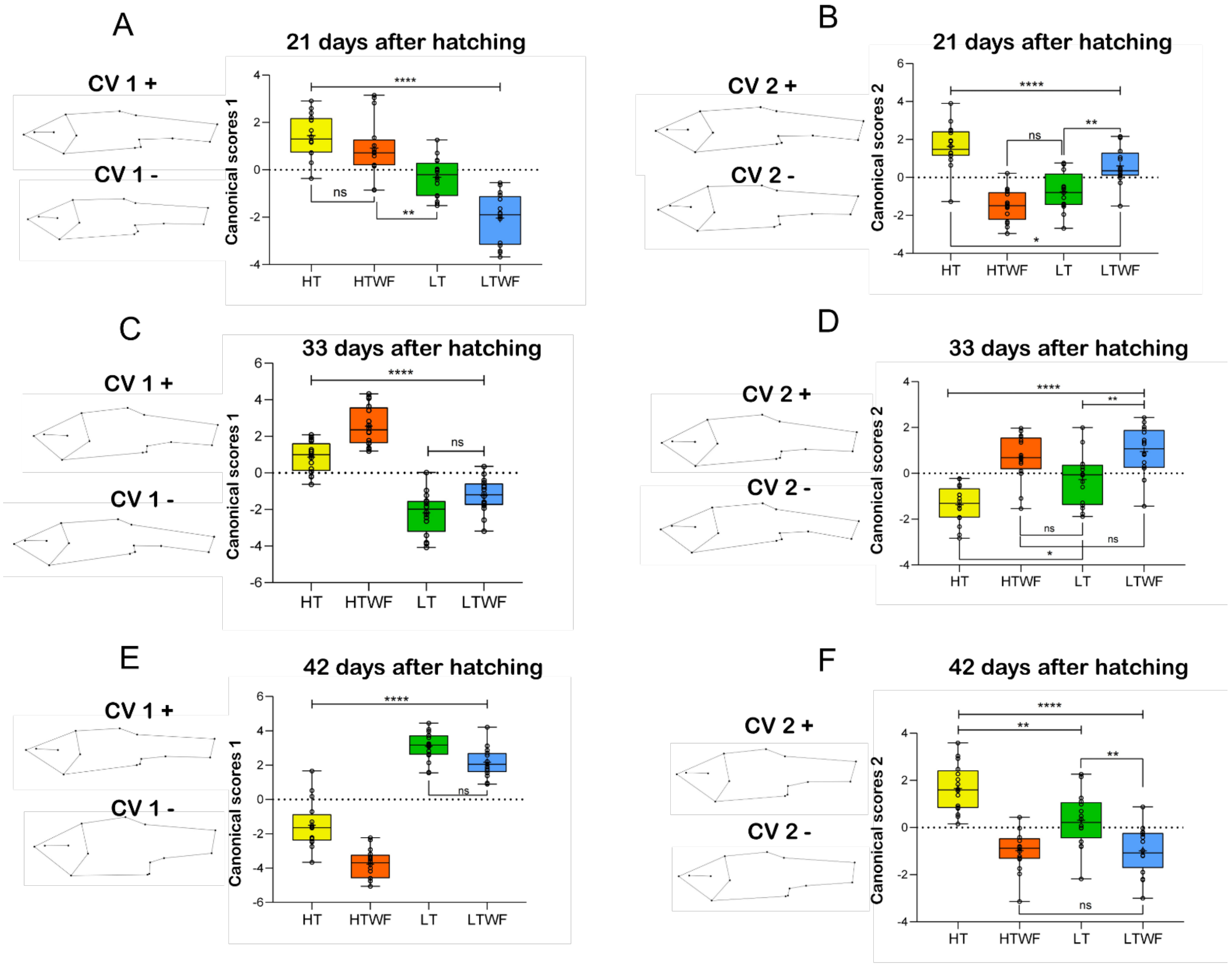

Supplementary Figure 4: Canonical Variate comparison between groups at 21, 33 and 42 days after hatching. A) 21 days after hatching: Canonical Variate 1, *p-values* HT-LT/HT-LTWF/LTWF-LT/HTWF-LTWF (****): < *0.0001*, HTWF/LT (**): *0.005*. B) 21 days after hatching: Canonical Variate 2, *p-values* HT-LT/HT-HTWF/HTWF-LTWF (****): < *0.0001*, (**) LTWF/LT: *0.001*, HT-LTWF (*): *0.03*0. C) 33 days after hatching: Canonical Variate 1, *p-values* HT-HTWFHT-LT/HT-LTWF/ HTWF-LTWF (****): < *0.0001*. D) 33 days after hatching: Canonical Variate 2, *p-values* HT-LTWF/HT-HTWF (****): < *0.0001*, (**) LTWF/LT: *0.006*, HT-LT (*): *0.018*. 42 days after hatching: Canonical Variate 1, *p-values* HT-HTWFHT-LT/HT-LTWF/ HTWF-LTWF (****): < *0.0001*. D) 42 days after hatching: Canonical Variate 2, *p-values* HT-LTWF/HT-HTWF (****): < *0.0001*, (**) HT-HTWF/LT: *0.003*, HT-LT (**): *0.002*. Mustard: HT (Hight Temperature), Orange: HTWF (Hight Temperature Water Flow), Green: LT (Low Temperature), Blue: LTWF (Low Temperature Water Flow)

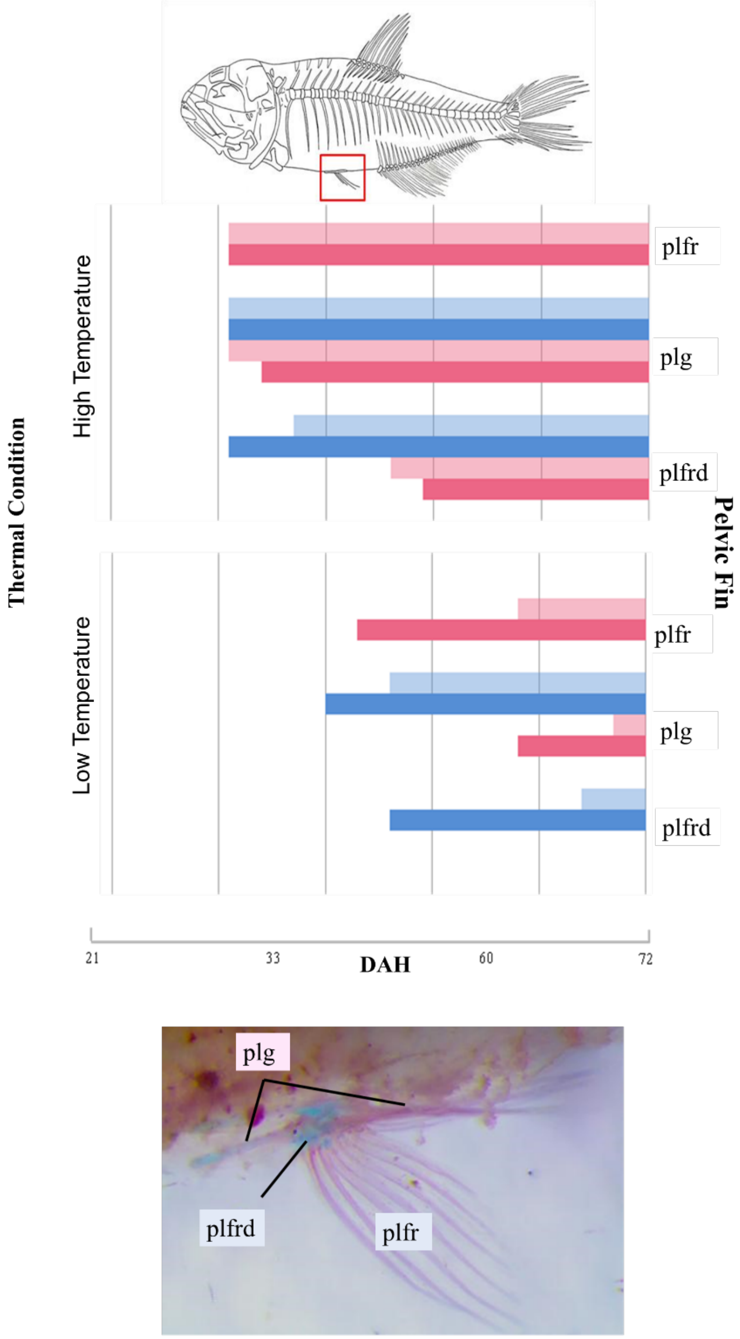

Supplementary Figure 5: chondrification and ossification sequence of skeletal elements from Pelvic fin: Pelvic Rays (plfr), Radials (plfrd) and Pelvic girdle (plg); Axis x: Days After Hatching (DAH), left axis y: High and Low Temperature, Right axis y: skeletal element and Fin. Blue bars: cartilage (Bright blue: No flow, Dark Blue: Flow presence), Red bars: bone (Bright red: No Flow, Dark red: Flow presence).

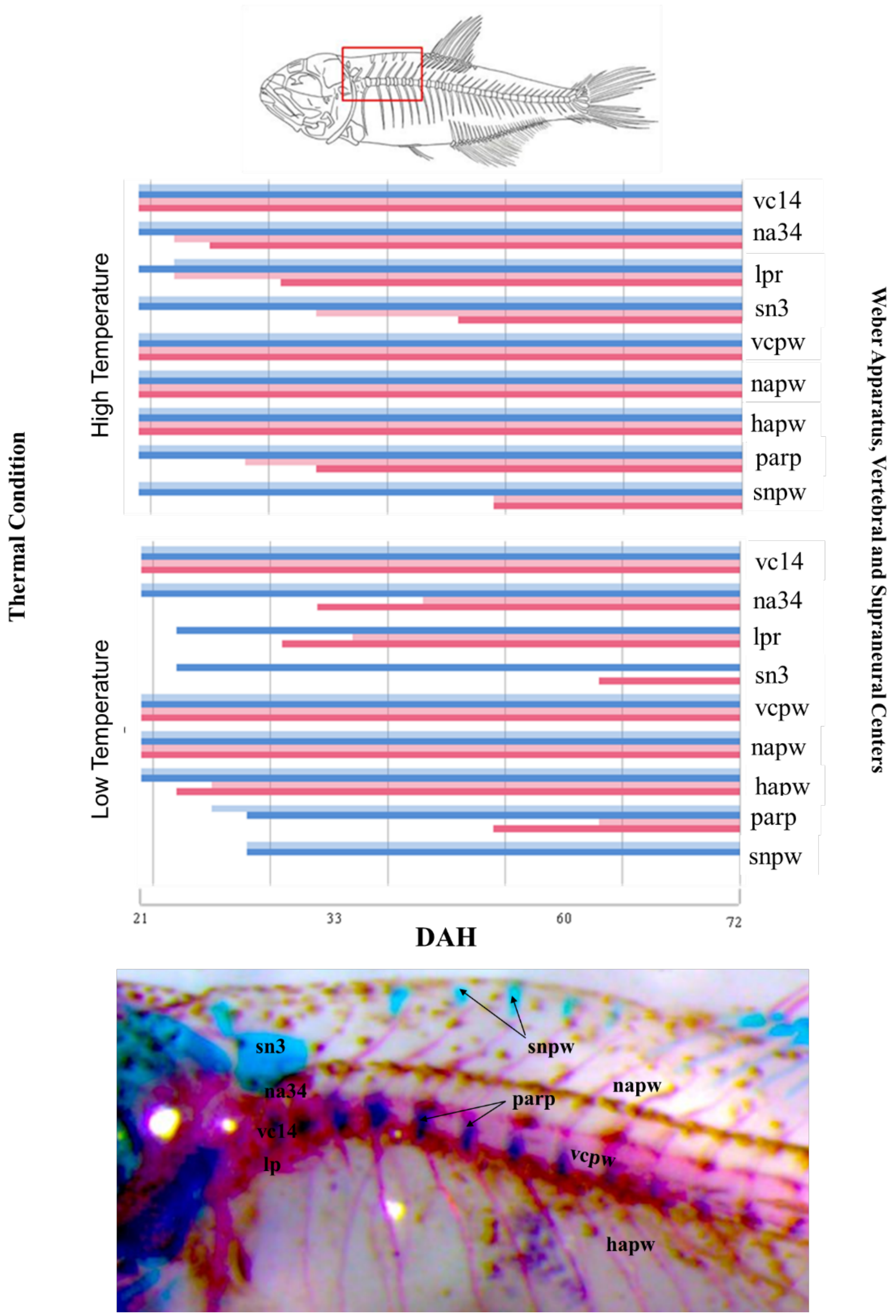

Supplementary Figure 6: chondrification and ossification sequence of skeletal elements from Weber apparatus, Vertebral and Supraneural centers: lateral processes (lpr), neural arches of vertebrae 3 and 4 (na34), vertebral centers 1, 2, 3 and 4 (vc14), and supraneural 3 (sn3), vertebral Centers (cvpw), Hemal Arches (hapw), Neural Arches (napw), Parapophyses (parp), Supraneural (snpw); Axis x: Days After Hatching (DAH), left axis y: High and Low Temperature, Right axis y: skeletal element and Fin. Blue bars: cartilage (Bright blue: No flow, Dark Blue: Flow presence), Red bars: bone (Bright red: No Flow, Dark red: Flow presence).

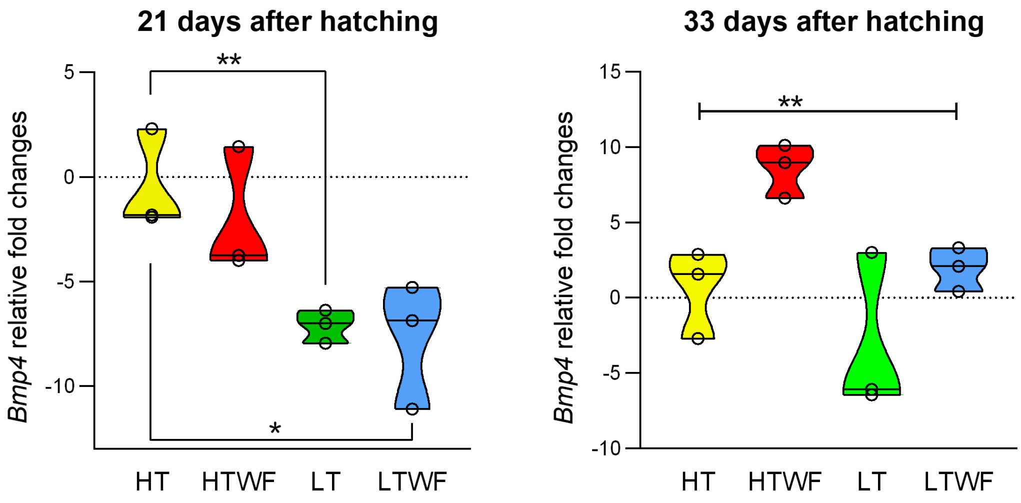

Supplementary Figure 7: *bmp4* expression levels comparison between experimental groups at 21 DAH (Left) and at 33 DAH (Right). 21 DAH: HT-LT (**) p-value: <0.001, HT-LTWF (*) p-value: <0.013. 33 DAH: HT-all groups (**) p-value: <0.009.

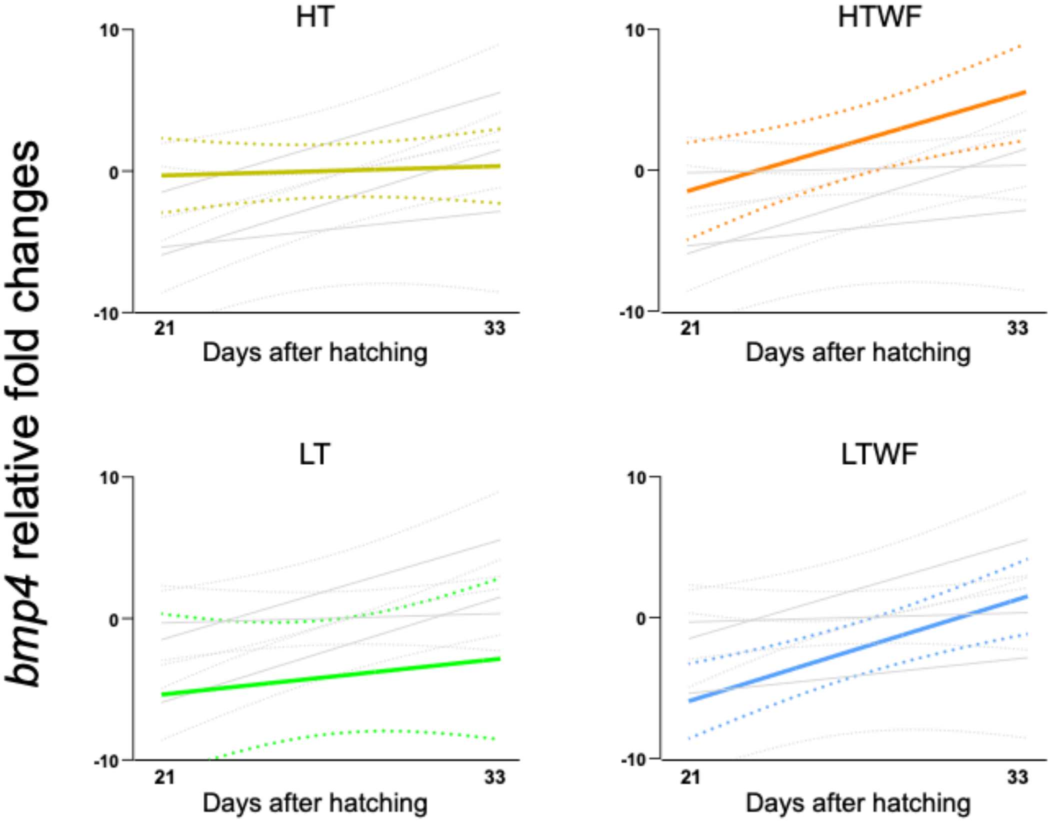

Supplementary Figure 8: Regression of *bmp4* expression levels in two sampling events (21dah and 33dah) shown separately for each experimental group: 1) HT = no water flow at high temperature, Rsquared = 0.056, F = 925 0.2382, p-value = 0.651; 2) HTWF = presence of water flow at high temperature, Rsquared = 0.870, F = 26.94, p-value = 0.006; 3) LT = no water flow at low temperature, Rsquared 926 = 0.279, F = 1.554, p-value = 0.280; 4) LTWF = presence of water flow at low temperature, Rsquared = 0.863, F = 25.29, p-value = 0.007.
