# Supplementary material for: Water temperature modulates multidimensional plastic responses to water flow during the ontogeny of a neotropical fish (Astyanax lacustris, characiformes)
Source: Front Cell Dev Biol. 2025 Jul 7;13:1531162. doi: 10.3389/fcell.2025.1531162 (PMC12277331; doi:10.3389/fcell.2025.1531162)
Supplement: Supplementary file 3 [file DataSheet1.pdf]

Supplementary Tables

Supplementary Table 1: Sampling design along the experiment.

SPECIMENS SAMPLING						
**COLLECTION**	**DAH***	**Data (month/day)**	**Morfologia**		**Molecular**	
**1**	21	01/17	4 especimens x 4 aquariums x 4 developmental conditions	64	4 especimens x 4 aquariums x 4 developmental conditions	32
**2**	24	01/20		64		32
**3**	27	01/23		64		32
**4**	30	01/26		64		32
**5**	33	01/29		64		32
**6**	36	02/01		64		32
**7**	39	02/04		64		32
**8**	42	02/07		64		32
**9**	45	02/10		64		32
**10**	48	02/13		64		32
**11**	51	02/16		64		32
**12**	54	02/19		64		32
**13-18**	57-72	02/22 to 03/09		96		-
	TOTAL:		**864**		**384**	

***Days After Hatching**

Supplementary Table 2: average body length (BL) by sampling day and experimental group.

DAH	AVERAGE BODY LENGTH (MM)			
	HT	HTWF	LT	LTWF
**21**	6.81 ± 0,43	6.99 ± 0,46	6.50 ± 0,36	6.77 ± 0,36
**24**	7.16 ± 0,46	7.51 ± 0,43	6.88 ± 0,43	7.12 ± 0,38
**27**	7.22 ± 0,74	7.48 ± 0,84	6.96 ± 0,61	6.92 ± 0,57
**30**	7.38 ± 1,08	7.83 ± 0,83	6.81 ± 0,60	7.24 ± 0,62
**33**	8.28 ± 1,26	9.08 ± 1,09	8.19 ± 0,45	8.67 ± 0,47
**36**	10.10 ± 1,13	11.50 ± 0,68	8.40 ± 0,50	8.97 ± 0,56
**39**	10.99 ± 1,30	12.24 ± 1,03	8.62 ± 0,48	9.10 ± 0,74
**42**	12.26 ± 1,43	13.69 ± 0,96	8.89 ± 0,39	9.75 ± 0,86
**45**	12.34 ± 1,92	14.33 ± 2,13	8.46 ± 0,62	9.42 ± 0,77
**48**	14.08 ± 2,89	15.80 ± 2,13	8.80 ± 0,69	10.10 ± 1,48
**51**	16.65 ± 3,66	18.76 ± 2,58	9.37 ± 0,66	10.60 ± 1,24
**54**	16.97 ± 3,47	21.19 ± 3,54	9.93 ± 0,47	11.22 ± 1,31
**57**	16.50 ± 1,72	-	10.62 ± 0,73	12.54 ± 1,97
**60**	18.39 ± 1,45	-	12.13 ± 0,57	12.84 ± 1,36
**63**	22.03 ± 0,95	-	13.12 ± 0,90	14.53 ± 1,64

Supplementary Table 3: Bonferroni's multiple comparisons test for body length (BL) to 21, 33, 42dah and Juvenile stage. Significant p-values are indicated in bold.

**Group comparison: 21 days**	***Degrees of freedom***	***t***	**Adjusted p value**
HT - HTWF	60	2.464	0.0990
HT - LT	60	2.194	0.1920
HT - LTWF	60	0.265	>0.999
HTWF - LT	60	4.658	**0.0001**
HTWF - LTWF	60	2.729	**0.0390**
LT - LTWF	60	1.929	0.3500
**Group comparison: 33 days**			
HT - HTWF	60	4.439	**0.0002**
HT - LT	60	0.201	>0.9990
HT - LTWF	60	1.428	0.9500
HTWF - LT	60	4.641	**0.0001**
HTWF - LTWF	60	3.011	**0.0220**
LT - LTWF	60	1.630	0.6500
**Group comparison: 42 days**			
HT - HTWF	60	4.562	**0.0002**
HT - LT	60	10.250	**<0.0001**
HT - LTWF	60	7.352	**<0.0001**
HTWF - LT	60	14.811	**<0.0001**
HTWF - LTWF	60	11.910	**<0.0001**
LT - LTWF	60	2.900	**0.0310**
**Group comparison: Juvenile**			
HT - HTWF	251	8.389	**<0.0001**
HT - LT	251	13.153	**<0.0001**
HT - LTWF	251	9.909	**<0.0001**
HTWF - LT	251	21.517	**<0.0001**
HTWF - LTWF	251	18.301	**<0.0001**
LT - LTWF	251	3.283	**0.0070**

Supplementary Table 4: Multivariate regression results. Significant p-values are indicated in bold.

**Test results**			
DF = 3	***F =*** 6.254	**p-value = 0.0004**	
**Group results**	**R squared**	**F**	**p-value**
**HT**	0.421	77.25	**<0.0001**
**HTWF**	0.488	101.1	**<0.0001**
**LT**	0.164	13.99	**0.0004**
**LTWF**	0.113	9.436	**0.0030**

Supplementary Table 5: Bonferroni's multiple comparisons test for Canonical Variate scores 1 and 2 (CV 1 and CV 2), which explain variation associated to Temperature and Water Flow, respectively. Groups: 21, 33, 42dah and Juvenile stage. Significant p-values are indicated in bold.

Groups: CV1 21 days	*Degrees of freedom*	*t*	Adjusted p-value
HT - HTWF	60	1.487	0.8530
HT - LT	60	4.973	**<0.0001**
HT - LTWF	60	9.834	**<0.0001**
HTWF - LT	60	3.486	**0.0050**
HTWF - LTWF	60	8.347	**<0.0001**
LT - LTWF	60	4.861	**<0.0001**
**Groups: CV2 21 days**			
HT - HTWF	60	8.824	**<0.0001**
HT - LT	60	6.755	**<0.0001**
HT - LTWF	60	2.907	**0.0300**
HTWF - LT	60	2.069	0.2570
HTWF - LTWF	60	5.917	**<0.0001**
LT - LTWF	60	3.848	**0.0010**
**Groups: CV1 33 days**			
HT - HTWF	60	4.771	**<0.0001**
HT - LT	60	8.641	**<0.0001**
HT - LTWF	60	5.953	**<0.0001**
HTWF - LT	60	13.410	**<0.0001**
HTWF - LTWF	60	10.720	**<0.0001**
LT - LTWF	60	2.688	0.05580
**Groups: CV2 33**			
HT - HTWF	60	5.818	**<0.0001**
HT - LT	60	3.095	**0.018**
HT - LTWF	60	6.550	**<0.0001**
HTWF - LT	60	2.724	0.0500
HTWF - LTWF	60	0.7318	>0.999
LT - LTWF	60	3.456	**0.006**
**Groups: CV1 42 days**			
HT - HTWF	60	6.375	**<0.0001**
HT - LT	60	13.030	**<0.0001**
HT - LTWF	60	10.390	**<0.0001**

HTWF - LT	60	19.416	**<0.0001**
HTWF - LTWF	60	16.772	**<0.0001**
LT - LTWF	60	2.642	0.0630
**Groups: CV2 42 days**			
HT - HTWF	60	7.456	**<0.0001**
HT - LT	60	3.807	**0.0020**
HT - LTWF	60	7.492	**<0.0001**
HTWF - LT	60	3.649	**0.003**
HTWF - LTWF	60	0.035	>0.9999
LT - LTWF	60	3.685	**0.0030**
**Groups: CV1 Juvenile**			
HT - HTWF	186	0.623	>0.999
HT - LT	186	16.866	**<0.0001**
HT - LTWF	186	15.181	**<0.0001**
HTWF - LT	186	17.352	**<0.0001**
HTWF - LTWF	186	15.701	**<0.0001**
LT - LTWF	186	2.042	0.2550
**Groups: CV2 Juvenile**			
HT - HTWF	186	10.730	**<0.0001**
HT - LT	186	0.188	>0.999
HT - LTWF	186	9.755	**<0.0001**
HTWF - LT	186	8.663	**<0.0001**
HTWF - LTWF	186	0.994	>0.999
LT - LTWF	186	8.403	**<0.0001**

Supplementary Table 6: Bonferroni's multiple comparisons test for differences in *bmp4* expression among groups. Groups: 21 and 33dah. Significant p-values are indicated in bold.

Group comparison: 21 days	*Degrees of freedom*	*t*	Adjusted p-value
HT - HTWF	8	1.043	>0.999
HT - LT	8	3.506	**0.008**
HT - LTWF	8	3.334	**0.013**
HTWF - LT	8	2.463	0.116
HTWF - LTWF	8	2.291	0.172
LT - LTWF	8	0.172	>0.999
**Group comparison: 33 days**			
HT - HTWF	8	4.043	**0.009**
HT - LT	8	1.797	0.490
HT - LTWF	8	0,731	>0.999
HTWF - LT	8	4.311	**0.003**
HTWF - LTWF	8	4.840	**0.001**
LT - LTWF	8	2.529	0.099

Supplementary Table 7: Regression results of *bmp4* expression as a function of days after hatching. Significant p-values are indicated in bold.

**Test results**			
**DF =** 3	**F =** 3.438	**p-value= 0.032**	
**Group results**	**R squared**	**F**	**p value**
**HT**	0.056	0.238	0.651
**HTWF**	0.870	26.94	**0.006**
**LT**	0.279	1.554	0.280
**LTWF**	0.863	25.290	**0.007**
